# Midterm Survivorship of an Uncemented Hydroxyapatite-Coated Titanium Femoral Component and Clinically Meaningful Outcomes in Patients Older Than 75 Years

**DOI:** 10.3390/jcm10051019

**Published:** 2021-03-02

**Authors:** Alexander Zimmerer, Luis Navas, Stefan Kinkel, Stefan Weiss, Matthias Hauschild, Marcus Streit

**Affiliations:** 1 ARCUS Sportklinik, Rastatterstr. 17-19, 75179 Pforzheim, Germany; navascontreras@sportklinik.de (L.N.); kinkel@sportklinik.de (S.K.); weiss@sportklinik.de (S.W.); hauschild@sportklinik.de (M.H.); Streit@sportklinik.de (M.S.); 2 Department of Orthopaedics and Orthopaedic Surgery, University Medicine Greifswald, Ferdinand-Sauerbruch-Straße, 17475 Greifswald, Germany

**Keywords:** Cementless THA, MCID, SCB, PASS, hip replacement, survival, old patient, octogenarians

## Abstract

Purpose: It remains controversial whether cementless femoral components are safe in elderly patients. The aim of this study was (1) to determine the stem survival rate in patients >75 years of age who were treated with an uncemented femoral component and (2) to report clinically significant results on a mid-term follow-up. Methods: 107 total hip arthroplasties (THA) were retrospectively evaluated in 97 patients over 75 years of age (mean age 78 years, range 75–87) treated with an uncemented femoral stem. The minimum follow-up was five years (mean 6.4 years, range 5–8). Stem survival rates, clinically meaningful outcomes, and incidence of complications were evaluated. Results: Kaplan-Meier survival analysis, with the endpoint revision for any reason, showed a 6.4-year survival rate of 98% (95% CI, 95–99%; 63 hips at risk). The survival rates were comparable for male and female patients (log-rank test, *p* = 0.58). The modified Harris Hip Score (mHHS) improved from 42.2 (12 to 85) points to 81.1 (22 to 97) points (*p* < 0.0001). Mid-term minimal clinically important difference (MCID), substantial clinical benefit (SCB), and patient acceptable symptomatic state (PASS) were 25, 84, and 70, respectively. Conclusion: An uncemented stem is a viable option in patients over 75 years with good clinical outcomes and survivorship. Periprosthetic fractures were not a relevant failure mechanism with the stem used.

## 1. Introduction

Total hip arthroplasty (THA) is one of the most successful orthopedic surgeries [1]. As life expectancy rises, there is an increased demand for total hip arthroplasty also in elderly patients. Cementless stem fixation has several advantages compared to cemented stem fixation, such as reduced operative time, reduced cardiopulmonary complications, and excellent long-term survival in several studies. Therefore, cementless fixation techniques gained popularity worldwide, especially in younger patients. Controversy persists regarding the safe use of uncemented stems in old patients, as some studies have shown an increased risk for periprosthetic fractures, especially in older female patients. In our center, we offer those patients with intraoperative sufficient bone stock for femoral fixation an uncemented THA regardless of their age.

The most recent annual report of the Australian Orthopaedic Association National Joint Replacement Registry (AOANJRR) indicated that cementless stem fixation had a higher revision rate in patients 75 years of age or older than cemented stem fixation [2]. Besides, the Norwegian Arthroplasty Register’s latest annual report indicated that women, in particular, have an increased risk of revision after cementless stem fixation [3]. However, these data represented a summary of a broad variety of cementless and cemented stems. Therefore, it is possible that the poorer results obtained with cementless stems simply reflect the number and performance of the individual stems used rather than the type of fixation. Nevertheless, the use of cementless fixation for older age groups has increased [4,5].

We, therefore, sought to (1) determine the survivorship using femoral revision for any reason as the endpoint, and in addition (2) report clinically meaningful outcomes in patients older than 75 years treated with one of the most used uncemented hydroxyapatite-coated titanium femoral components at mid-term follow-up.

## 2. Methods

### 2.1. Patient Selection

We retrospectively evaluated the clinical results of a multi-surgeon series of 107 THAs performed in 97 patients older than 75 years that underwent surgery between January 2012 and December 2014 at our institution with uncemented hydroxyapatite (HA)-coated titanium femoral stems (Corail stem, DePuy Orthopaedics, Warsaw, Indiana, USA). The Corail standard stem is a tapered grit-blasted stem and coated with a 150-μm thick layer of hydroxyapatite. The FDA cleared this femoral component for use in the United States. We use cementless stems in patients without severe femoral canal deformity and adequate bone stock for uncemented femoral fixation, determined intraoperatively. The achievement of a rotation-stable cementless fixation of the stem was considered to be adequate bone stock. In the same period, 13 patients older than 75 years received a cemented femoral component due to inadequate bone stock.

Surgery was performed on 39 men and 58 women. The diagnoses leading to arthroplasty were osteoarthrosis in 89 and congenital dysplasia of the hip (CDH) in 18 hips. The mean body mass index at surgery was 26.6 kg/m^2^ (range, 20.2 to 41.8 kg/m^2^). The mean age at surgery was 78 years (range, 75 to 87 years) (Table 1). Six patients had died at a mean follow-up of 6.4 years (range, 5 to 8), and seven were lost to follow-up.

A minimal-invasive posterior approach was used in 33 hips, a modified direct lateral approach in 46 patients, and a direct anterior approach (DAA) in 28 patients according to the expertise of the performing surgeon. Recommendations for return to activities following THA met the consensus guidelines from members of the Hip Society and the American Association of Hip and Knee Surgeons [6].

A cementless (HA)-coated titanium Corail stem was used in all hips, and 89 (83%) femoral stems had a collar. Collared designs are thought to enhance primary stability by improving resistance to axial, rotational, and varus forces at the bone-implant interface [7]. A cementless Allofit acetabular cup (Zimmer, Warsaw, IN, USA) was used in 8 hips, and a Pinnacle acetabular cup (Depuy Orthopaedics, Warsaw, IN, USA) in 98 hips, and one patient received a cemented Triloc cup (Depuy Orthopaedics, Warsaw, IN, USA) respectively. Ten patients received bilateral THA.

The ethics commission (Ethikkommission der Landesärztekammer Baden-Württemberg Germany, F-2019-006) approved all procedures, and the study was conducted in accordance with the Helsinki Declaration of 1975, as revised in 2008 [8]. All patients gave informed consent.

### 2.2. Psychometric Analysis

As a matter of routine, the modified Harris Hip Score (mHHS) [9,10] was assessed preoperatively and at the latest follow-up in our institutional joint arthroplasty registry. Patients reported a visual analog scale (VAS) for pain at these points and VAS for satisfaction at the latest follow-up. The scores were obtained using a questionnaire. At the latest follow-up, the data of 80 patients (90 hips) were available.

To quantify the clinical significance of the meaningful outcome achievement, the minimal clinically important difference (MCID), the substantial clinical benefit (SCB), and the patient acceptable symptomatic state (PASS) were calculated for mHHS.

MCID was determined by calculating an anchor-based method [11]. At the latest follow-up, patients were asked the following anchor question: “Since your total hip arthroplasty, how would you rate your overall physical ability?” Patients’ answer choices were “much worse,” “worse,” “slightly worse,” “no change,” “slightly improved,” ‘‘improved,’’ and ‘‘much improved.’’ To define minimal improvement, we considered the difference between those who answered “improved” (experienced minimal improvement) to those who reported “slightly improved” or “no change” (did not experience minimal improvement).

The absolute SCB was calculated using an anchor-based method [12]. At the latest follow-up, patients were asked the following anchor question: ‘‘Since your total hip arthroplasty, how would you rate your overall physical ability?’’ Patients’ answer choices were ‘‘much worse,’’ ‘‘worse,’’ ‘‘slightly worse,’’ ‘‘no change,’’ ‘‘slightly improved,’’ ‘‘improved,’’ and ‘‘much improved.’’ Patients who responded with “slightly worse”, “no change”, or “slightly improved” were used as a control group. The corresponding difference between the control group and ‘‘much improved’’ was used to define the SCB.

PASS was calculated by the use of an anchor-based method. Patients were asked the following question at the latest follow-up [13,14]: ‘‘Taking into account all the activities you have during your daily life, your level of pain, and also your functional impairment, do you consider that your current state is satisfactory?’’ The MCID, SCB, and PASS for mHHS were calculated using a receiver operating characteristic (ROC) curve analysis [15], an area under the curve > 0.8 was considered predictive of patients who did and did not achieve MCID, SCB, and PASS. The cutoff point was defined using Youden’s Index [16].

### 2.3. Statistics and Survival Analysis

Kaplan-Meier survivorship analysis was performed with the use of revision for any reason as the endpoint. A revision was defined as “an operation that involved removing and/or replacing one or more components of a joint replacement.” Differences in survival rates were tested for statistical significance using the two-sided log-rank test. Continuous variables were compared with the use of a two-sided Student’s *t*-test. We considered *p*-values of <0.05 to be significant. Statistical analyses were conducted using XLstat statistics software (ADDINSOFT, Paris, France).

## 3. Results

### 3.1. Demographics

**Figure 1:**
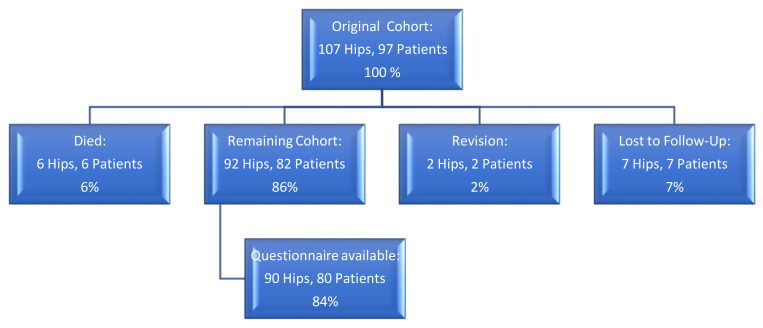
Flowchart illustrating Patient status at most recent follow-up.

At a mean follow-up of 6.4 years (range, 5–8), six patients (six hips) had died without a revision prior to death for reasons unrelated to the surgery; the mean time between operation and death was five years (range, 2 to 6.3 years). Seven patients (seven hips) were lost to follow-up (these patients had moved and could not be followed). In two hips (two patients), a revision was performed (Figure 1). The remaining 92 hips in 82 patients were available for review at final follow-up. The clinical questionnaire was available for 80 patients (90 hips). Two patients (2 hips) had only a telephone interview regarding the survival and revision status as they were unwilling to participate in the questionnaire.

### 3.2. Survival Analysis

**Figure 2:**
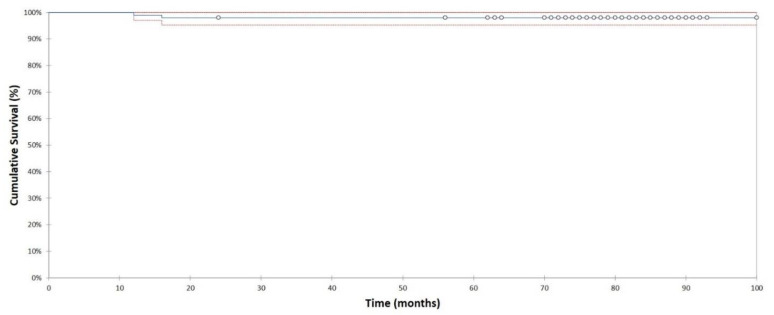
Kaplan-Meier survivorship curve and 95% confidence intervals with revision for any reason as the endpoint. 6.3-year survival was estimated at 98% (95% CI, 55–99%; 63 hips at risk).

Two out of 107 total hip arthroplasties (2%) have been revised. In one hip, the stem and the cup were removed for deep infection 1.3 years after the index surgery. In another hip, an isolated revision of the stem was performed because of stem migration due to an undersizing. In this case, a too-small uncollared stem was implanted, which migrated during loading. Both revisions were male hips. The Kaplan-Meier survival analysis, with the endpoint being femoral revision for any reason, estimated the 6.4-year survival rate at 98% (95% CI, 95–99%; 63 hips at risk; Figure 2).

**Figure 3:**
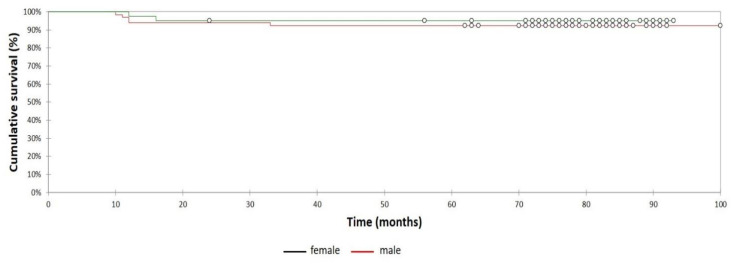
Kaplan-Meier survivorship curve and 95% confidence intervals with revision for any reason as the endpoint according to gender. 6.3-year survival was estimated at 95% (95% CI, 88–99%; 63 hips at risk) for male patients and 100% ([95% CI, 86–99%; 39 hips at risk) for female patients. (Log rank test, *p* = 0.58).

When the seven patients lost to follow-up were considered as revised, worst case survival with the endpoint revision for any reason was 93% (95% CI, 83–91%; 63 hips at risk) at 6.3 years. The 6.4 years survival rates with the endpoint being revision for any reason were comparable for male (95% [95% CI, 88–99%; 22 hips at risk]) and female (100% [95% CI, 86–99%; 39 hips at risk]) patients (Figure 3). The difference was statistically not significant (Log-rank test, *p*  =  0.58).

Table 2 shows available survival data on uncemented femoral components in patients older than 75 years.

A post-operative hematoma requiring surgery occurred in 5 patients (5 hips). One female patient (1 hip, posterior approach) developed a post-operative abductor deficiency. One female patient (1 hip) had the femoral head component revised due to component incompatibility. No periprosthetic fractures were documented for the overall study cohort.

### 3.3. Analysis of Pre- Versus Postoperative Reported Outcome Score Measurements

Analysis of preoperative and midterm follow-up reported mHHS demonstrated statistically significant improvement. The mHHS improved from 42.2 (12 to 85) points preoperatively to 81.1 (22 to 97) points post-operatively (*p* < 0.0001). Besides, there was a statistically significant improvement in pain VAS (9.0 (4–10) vs. 1.5 (0–5) points (*p* < 0.0001). The average VAS for satisfaction at follow-up time was 9.1 (2 to 10).

### 3.4. Achievement MCID, SCB, and PASS

The mHHS midterm threshold scores for achieving MCID, SCB, and PASS were 25, 84 and 70, respectively. A total of 67 (74%) patients achieved MCID mHHS, 49 (54%) achieved SCB mHHS, and 74 (81%) achieved PASS mHHS, respectively.

## 4. Discussion

The present study’s main finding was a 6.4-year survival rate of 98% after implanting an uncemented HA-coated stem in patients >75 years of age. No periprosthetic fracture was diagnosed in the investigated cohort, so this complication was not a relevant failure mechanism. Besides, the present analysis reported clinically significant results with the MCID, SCB, and PASS as a new method of PROM evaluation for the mHHS.

Cementless femoral component use in primary THA has significantly increased over the last decades. [22,23,24,25] Among the main risks for periprosthetic fractures in cementless THA, higher patient age has been identified. [21] In contrast, cemented femoral stems have been shown to minimize the risk of early complications with excellent long-term survivorship. [5,26,27] Therefore, cemented THA was predominantly used in older patients. [2,3,28] However, there are complications even with cemented femoral stems, such as bone cement implantation syndrome (BCIS) [29,30,31,32,33] and prolonged operative time. To prevent these complications, cementless THAs have been increasingly used in older patients in recent years.

Few data on the outcome of patients 75 years and older after cementless THA are currently available in the literature. Besides the results of the register studies, which attribute a favorable long-term outcome to the cemented stems, recent studies have collectively emphasized that the use of cementless fixation is feasible in older patients [4,17,18,19,20,21,34]. These studies showed low revision rates and a high survival rate in mid-term follow-up, which the results of our study confirmed. An explanation for varying results in register data could be that these data represent a summary of a wide variety of cementless and cemented stems. Therefore, it is possible that the inferior results obtained with cementless stems simply reflect the number and performance of the individual stems used rather than the type of fixation. One possible explanation for the different results may be the implantation technique. While an impaction broaching system is used for the Corail stem, an extraction broaching system is used for many other stems. However, further studies are needed to investigate these controversial results.

Female gender was identified as a risk for revision of a cementless stem in recent publications. [3,26] As both patients in our collective were male, we cannot confirm this risk factor. However, it must be noted that our sample had a small number of cases and, therefore, might be underpowered to detect a difference. While aseptic loosening is the leading cause of implant failure in younger patients, periprosthetic fracture seems to be the most common cause of failure in older people with cementless stem fixation. [21] Recent research has shown that using a calcar collar can significantly reduce the risk of periprosthetic fracture. [35] Furthermore, it has been shown that anatomical stem designs have been associated with a lower risk of periprosthetic fracture compared with tapered designs in cementless stems. [36,37] However, a taper-designed stem was used in the present study, and no periprosthetic fracture was observed.

In addition to the survival analyses, PROMs were collected and evaluated. We demonstrated a significant improvement in mHHS, which was 81.1 points on average at mid-term follow-up. These results are consistent with those reported in the literature for THA in elderly patients. [17,38,39] The MCID, SCB, and PASS were used to determine meaningful clinical outcomes. The reporting of meaningful clinical outcomes is relatively new in endoprosthetics and is considerably more regularly reported in hip joint preserving surgery [13,40,41,42,43,44,45]. For this reason, there is little data available to compare our findings. It is essential to define these new parameters for the various patient groups and follow-up times to provide valuable information for future studies and clarify which results patients consider satisfactory.

Our study is not free of limitations. First of all, there is a small study population, so that the influence of possible risk factors for THA revision, such as female gender or the presence of osteoporosis, cannot be sufficiently assessed. Furthermore, the patients were only examined by questionnaire at the final follow-up and not clinically or radiologically. This may lead to the fact that a failure like a migration of the stem could not be detected in asymptomatic patients. Furthermore, our study design does not include a control group that was younger or received a cemented femoral component. A comparison of the results with a control group was thus not possible. Besides, many patients underwent implantation of a collared stem, so that the conclusion can only be attributed to this stem and cannot be generalized.

## 5. Conclusions

The study shows a 6.3-year survival rate of 98% after implanting an uncemented HA-coated stem in patients > 75 years of age with good clinical outcomes. The survival rate in this patient cohort is consistent with literature results from younger patients. The cementless stem used shows a low revision rate even in patients >75 years of age over the mid-term. Periprosthetic fractures were not a relevant failure mechanism.

## Figures and Tables

**Table 1:** Patient Demographic Data.

	Male	Female	Total
Age years, mean (range)	78 (75 to 87)	78 (75 to 84)	78 (75 to 87)
Hips, no. (%)	41 (38)	66 (42)	107
Body mass index kg/m^2^, mean (range)	26.7 (21.3 to 33.6)	26.6 (20.2 to 41.8)	26.6 (20.2 to 41.8)
Right:Left	19:22	37:29	56:51

**Table 2:** Overview of the reported survival rates of uncemented femoral components in patients > 75 years.

Authors	Year of Publication	Sample Size	Mean Follow-Up	Stem(s) Used	Stem Survival overall %	Level of Evidence
Yuasa et al. [17]	2016	30	5.6 years	- Natural Hip (Zimmer, Warsaw, Indiana, USA) - Synergy (Smith&Nephew, London, UK)	100%	4
Riley et al. [18]	2016	78	Minimum 2 years	- Accolade I or Accolade II (Stryker, Mahwah, NJ, USA)	98.7%	3
Stihsen et al. [19]	2017	162	10.7 years	- Zweymüller SL, SLL or SLO (Zimmer, Warsaw, Indiana, USA)	100%	3
Ahmad et al. [20]	2018	76	Minimum 5 years	- Polar (Smith&Nephew, London, UK)	97.4%	3
Gkagkalis et al. [21]	2019	121	4.1 years	- Optimys (Mathys Ltd., Bettlach, Switzerland)	100%	3
Current study		107	6.4 years	- Corail (DePuy Orthopaedics, Warsaw, Indiana, USA)	98%	3
